# A Sensor-Based Multichannel FES System to Control Knee Joint and Reduce Stance Phase Asymmetry in Post-Stroke Gait

**DOI:** 10.3390/s21062134

**Published:** 2021-03-18

**Authors:** Benoît Sijobert, Christine Azevedo, Joanna Pontier, Sahara Graf, Charles Fattal

**Affiliations:** 1 Institut Saint-Pierre, 34250 Palavas, France; 2 INRIA, Sophia-Antipolis, 06902 Montpellier, France; christine.azevedo@inria.fr; 3 CRF La Châtaigneraie, 95180 Menucourt, France; jpontier@gmail.com; 4 GHICL, 59462 Lomme, France; graf.sahara@ghicl.net; 5 USSAP, 66962 Perpignan, France; cfattal@ussap.fr

**Keywords:** stroke, neuro-rehabilitation, motion analysis, functional electrical stimulation

## Abstract

Most of the studies using functional electrical stimulation (FES) in gait rehabilitation have been focused on correcting the drop foot syndrome. Using FES to control the knee joint in individuals with central nervous system (CNS) disorders could also play a key role in gait recovery: spasticity decrease, higher range of motion, positive effect on balance, limiting hyperextension and flexion in stance phase, reducing joint overload, etc. In stance phase, an accurate timing and a fine tuning of stimulation parameters are however required to provide a proper control of the knee stimulation while ensuring a safe and efficient support. In this study, 11 participants were equipped with inertial measurements units (IMU) and foot pressure insoles after supratentorial ischemic or hemorrhagic stroke, informing on knee angle and gait events used to online adapt FES during a 10 m walking protocol. Asymmetry of stance time and weight bearing were monitored as well as gait quality and physiological cost through a series of relevant markers. Vertical trunk motion has been significantly reduced during gait with FES (*p*-value = 0.038). Despite no significant improvement of stance phase asymmetry has been found, this preliminary work shows evidence of promising technical and rehabilitative potentials of a sensor-based multichannel FES system to control knee joint in post-stroke gait.

## 1. Introduction

Numerous strategies using functional electrical stimulation (FES) have been investigated over the past fifty years to assist or restore gait [1]. Most of these studies have been conducted in post-stroke individuals and have focused on correcting the drop foot syndrome by supplementing the absence of dorsiflexion [2]. The principle of drop foot stimulation (DFS) is to activate foot dorsiflexion and eversion from heel off to heel on events by stimulating common peroneal (CP) nerve and/or tibialis anterior muscle. The state-of-the-art reflects a substantial lack of interest in using FES to improve knee joint control in central nervous system (CNS) disorders, where this problematic is classically handled by using a knee orthosis. However, focusing on this specific joint could play a key role in post-stroke gait recovery. Indeed, in hemiplegic individuals, using FES on a stiff knee has proven to decrease spasticity of the knee flexors and extensors and increase their range of motion [3,4]. A preliminary evidence of a positive therapeutic effect on balance and mobility was also observed using FES-based knee control in early stroke rehabilitation [5,6]. Multiple studies suggested that preventing hyperextension (genu recurvatum) and enabling a small knee flexion of the paretic limb during the stance phase would be helpful to improve gait recovery. During stance phase, the knee flexion is lower than 10° in able bodied individuals when walking at a slow gait pace, which is similar to a FES assisted walk (<0.5 m/s) [7]. Meanwhile, in diplegic gait the individuals walk with their knees considerably flexed. This crouch gait leads to an important joint overload. Perry et al. [8] showed that a knee flexed at an angle of 30° requires that the quadriceps ensures a force to stabilize the knee equal to 210% of the load on the femoral head, while a flexion of 15° decreased this force to 75% of the load. Chantraine et al. [9] proposed to use an implanted DFS and to extend the timing of the CP nerve stimulation during the stance phase in order to tilt the tibia forward. This appeared to effectively limit the knee hyperextension, with continued positive outcomes more than 12 months following implantation. Another study also demonstrated that to prematurely stimulate the quadriceps just after IC (Initial Contact) could also lead to a knee hyperextension thereby preventing shock absorption by a too early joint locking [10]. During mid-stance, stiff limb restricted to the sagittal plane by the use of a knee orthosis (knee brace) creates a compass type gait causing excessive vertical center of mass motion and requiring excessive effort to carry the body over the stance limb [10]. A stiff knee at the end of the stance phase prevents from easily going forward in the swing phase. Reinbolt et al. study [11] showed that a late deactivation of the knee extensors decreases the maximum knee flexion angle in swing phase. Meanwhile, in the absence of voluntary control, Kobetic et al. [12] observed that a premature deactivation of the knee extensors at the end of the stance phase before the leg is fully unloaded can lead to a fall.

These observations reflect the importance and the need of an appropriate and accurate timing in the control of the thigh muscles stimulation in stance phase while ensuring a safe and efficient support.

Different thigh muscle activations could be used on individuals with a CNS disorder in order to correct an identified knee problem (e.g., crouch gait, stiff knee, genu recurvatum, etc) throughout the gait cycle [13,14]. Being able to adapt the assistive control to multiple gait patterns and compensatory strategies also requires to accurately monitor the knee angle in swing and stance phases. This information highlights, in addition to an accurate timing and knee angle monitoring, the need of studying a solution able to provide an individualized and specific control of the stimulation depending on the patient’s gait features.

Based on promising preliminary evidence from literature, the potential of this neurorehabilitation paradigm motivated us to further investigate assistive closed-loop control of the knee in post-stroke subjects. The use of inertial measurement units to assess pathological gait and of the knowledge addressed in previous studies [15,16] has enabled us to go further and to face the multiple constraints of an online closed-loop stimulation protocol in this specific context. This preliminary study aims to investigate the technical and rehabilitative potentials of our approach through the use of a sensor-based multichannel FES system to control knee joint and reduce stance phase asymmetry in post-stroke gait.

## 2. Materials and Methods

### 2.1. Instrumentation

**Figure 1:**
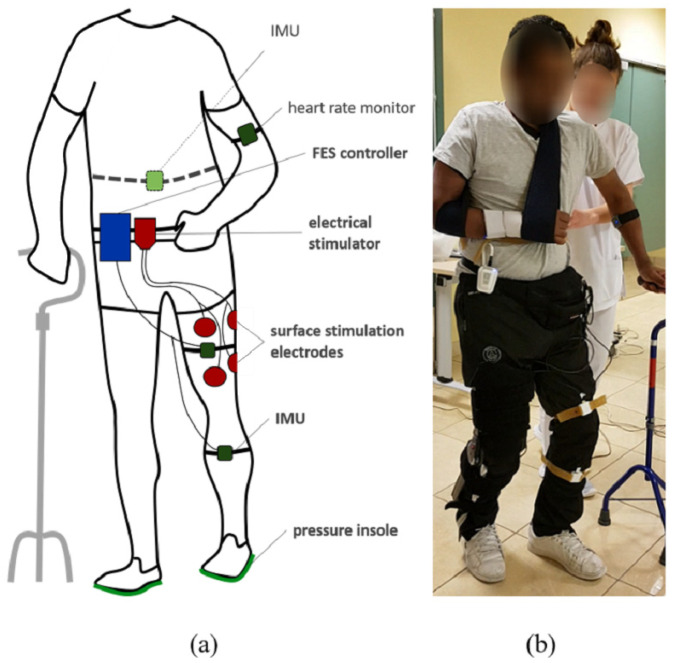
Experimental setup diagram (**a**) and associated picture (**b**). The participants are equipped with pressure insoles and two IMUs on the leg. A controller records and processes data to adapt online FES of quadriceps and hamstrings depending on knee angle and gait events. A wireless IMU in the back and a heart rate sensor are additionally used to evaluate motion and performances.

To monitor weight bearing and stance time asymmetry, participants were equipped with bluetooth instrumented insoles (FeetMe©, Versailles, France) able to measure at a 100 Hz sampling rate the pressure distribution through a matrix of sensors. The subjects were also equipped with two inertial measurement units (IMU Bosch© BNO055, Gerlingen, Germany) located at the thigh and the tibia by means of rubber straps (Figure 1). IMUs were wired to a Raspberry Pi3© control unit. Each IMU embedded a high speed ARM Cortex-M0 based processor and a Kalman Filter directly providing the quaternion estimation needed to compute knee angles at a 100 Hz sampling rate, using a goniometer computation published in a previous work [15]. Stimulation was sent via a two-channel wireless stimulator (Phenix Neo©, Montpellier, France) to the quadriceps and hamstrings via rectangular surface electrodes (50 × 90 mm). One wireless IMU (Fox HikoB©, Villeurbanne, France) was installed in the back of the participants at the second sacral vertebra level to estimate vertical trunk displacement. An armband heart rate monitor (Scosch Rhythm©, Oxnard, CA, USA) was set up on each participant to record cardiac frequency in order to compute the physiological cost index (PCI) (Figure 1). All data was synchronously recorded using a multi-threading script programmed in Python and executed onto the Raspberry Pi3© control unit.

### 2.2. Control Modality

#### 2.2.1. Knee Control

Initially, a knee angle setpoint (KAS) was defined by the practitioner as the optimal knee flexion during stance phase (demonstrated to be around 5° of flexion [17]). The stance phase was detected through the foot pressure insoles. The use of an onset threshold applied to the insole data has been demonstrated to be reliable enough to determine the beginning and end of the stance phase [18]. Based on a real-time pressure mapping of the feet, the stance phase was similarly estimated in our study as a threshold crossing of the sum of pressure matrices given by the insoles. The threshold was settled to be the same for all the subjects and expressed without unit. For each subject, a calibration was performed with the feet up in the air in order to eliminate possible residual pressures due to the tightening of the laces.

Stimulation was sent either to quadriceps or hamstrings, depending on the actual paretic knee angle (PKA) compared to the desired KAS in stance phase. A knee too flexed led to the closed-loop stimulation of the quadriceps, while a knee too extended led to the stimulation of the hamstrings.

**Figure 2:**
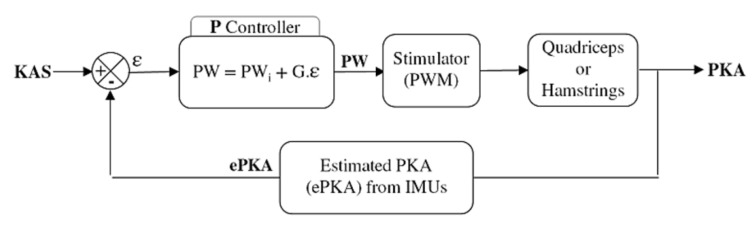
Closed-loop control of the thigh muscles. A P controller modulates the pulse width of the stimulator depending on the error between the knee angle setpoint (KAS) and the estimated paretic knee angle (ePKA) from the IMUs quaternions during stance phase. An initial pulse width (*PWi*) and a gain G are set for each participant depending on his knee issue.

An initial pulse width value *PW_i_* was defined for each participant and each muscle (quadriceps and hamstrings) as the first value to elicit an efficient motor response of the muscle. This was firstly determined while the subject was seated. The practitioner checked visually and with his hand each stimulated muscle. The experimenter rised the pulsewidth until a slight contraction could be detected. From this point, the participant was asked to stand up and the practitioner manually assessed the efficiency of the stimulation level to lock the knee, and if needed the *PW_i_* value was increased. This initial stimulation level was used (1) as a pre-stance stimulation, to lock the knee before initial contact; (2) as the initial level of stimulation used in the proportional (P) controller of the closed-loop (Figure 2). Once the frequency and the intensity of the stimulation have been set (i.e., f = 30 Hz, I = 50 mA), only the pulse width was modulated. The P controller adjusted the pulse width depending on the error ε between the estimated PKA (ePKA), computed from the IMUs quaternions, and the desired KAS.

A maximum pulse width *PW_max_* was determined as the maximum bearable level of the stimulation before pain. A maximum range of motion *ROM_max_* was defined in extension (hamstrings) and in flexion (quadriceps) around the KAS (e.g., KAS-*ROM_max-quadri_* < KAS < KAS + *ROM_max-hamstrings_*).

The controller gain *G* was automatically computed for each participant and each muscle group depending on *PW_i_*, *PW_max_* and *ROM_max_* in order to bind the stimulation pulse width to *PW_max_* when the maximum range of motion was reached:

(1) $$G=\frac{PW_{max}-PW_{i}}{ROM_{max}}$$

**Figure 3:**
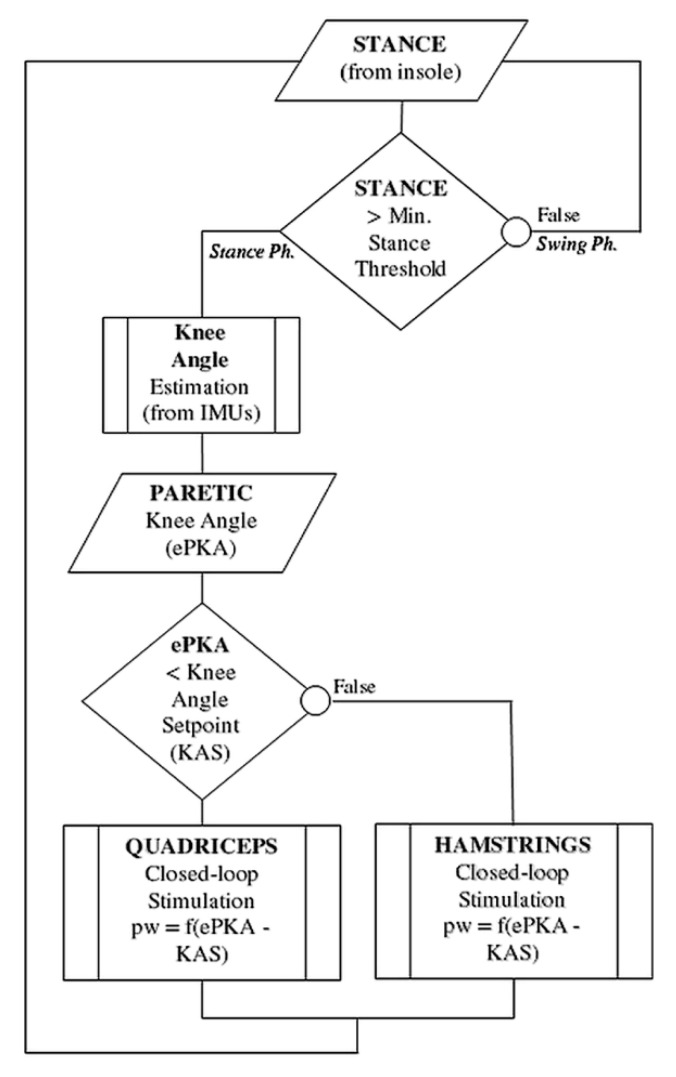
Flowchart of the IF-THEN rules regulating online the stimulation control. The stance phase is detected using a simple threshold on the foot pressure insoles. Stimulation is sent either to quadriceps or hamstrings, depending on the actual paretic knee angle (PKA) compared to the desired KAS (Knee Angle Setpoint) in stance phase. A knee too flexed led to the closed-loop control of the quadriceps, while a knee too extended led to the control of the hamstrings.

The flowchart in Figure 3 summarizes the IF-THEN rules regulating knee stimulation throughout the control cycle.

#### 2.2.2. Pre-Stance Event

As mentioned previously, a CNS disorder can be associated with different impairments at the knee level. An increased knee flexion in stance phase is a consequence of a crouch gait, while a genu recurvatum leads to a hyper-extension. To compensate the motor response delay and eventually counteract a hyper-flexed or hyper-extended knee before the stance phase, we integrated in the control modalities the possibility of triggering a pre-stance stimulation.

Multiple studies investigated the delay between the muscle force response and the time of electrical stimulation onset [19]. The order of the magnitude usually considered was around 100 ms [20,21]. In addition, the global hardware latency was added to this physiological latency in order to take into account the delays between the actual stimulation event (e.g.,the foot reached the ground), the time to process data, the detection algorithm, and the triggering of the stimulator.

In severe cases of crouch gait or genu recurvatum, starting the control of the stimulation on the detection of IC (Initial Contact) might be too late to reach an efficient motor response on time and correct the knee problem over the stance phase.

**Figure 4:**
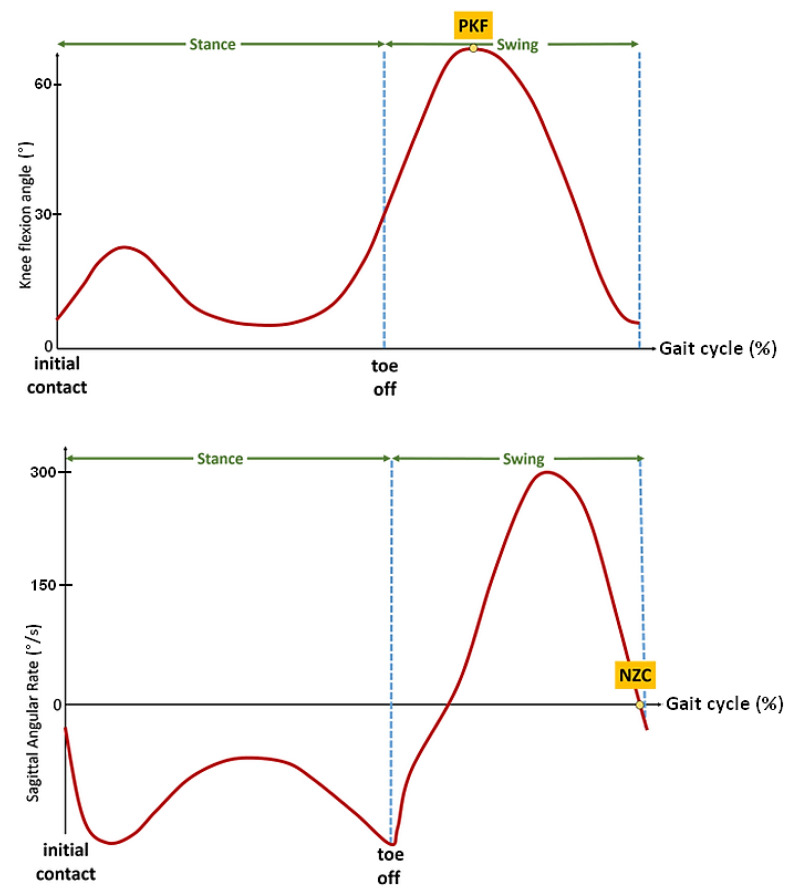
Illustrative figures of the two events to be detected before stance phase. Top: Peak knee flexion (PKF) angle in swing phase, Bottom: negative zero crossing (NZC) sagittal angular rate.

To minimize the motor response delay and obtain a rapid and forceful response, Andrews et al. [22] applied a relatively high frequency stimulation (up to 100 Hz) to the thigh muscles to progressively reduce the frequency to 20 Hz with an automatic compensation of pulse width. The stimulator used in our protocol did not enable us to online modulate the frequency and apply this strategy. Therefore, a pre-stance event detection algorithm was studied and developed in order to be able to anticipate the stance phase in some participants with a specific pathological gait. Inspired by previous studies led by the authors [23,24], two main events have seemed to be relevant and easily detectable in real time on these heavily impaired gait patterns: the peak knee flexion (PKF) angle during swing phase and the negative zero crossing (NZC) of the sagittal angular speed recorded from the gyrometer output signal located on the tibia (Figure 4).

Used in gait assessment [6] to reliably detect the gait cycle from inertial data, the NZC corresponds to an event occurring right before termination of forward swing. As observed in previous work on post-stroke gait [15], this gyrometer characteristic waveform is in most of the cases still present in highly impaired gait. In addition, the NZC detection algorithm is easily implementable by monitoring the sign and magnitude of the angular rate in the swing phase. Above a minimum motion threshold, if two consecutive gyrometer samples changed from a positive to a negative sign, the zero is considered as crossed.

**Figure 5:**
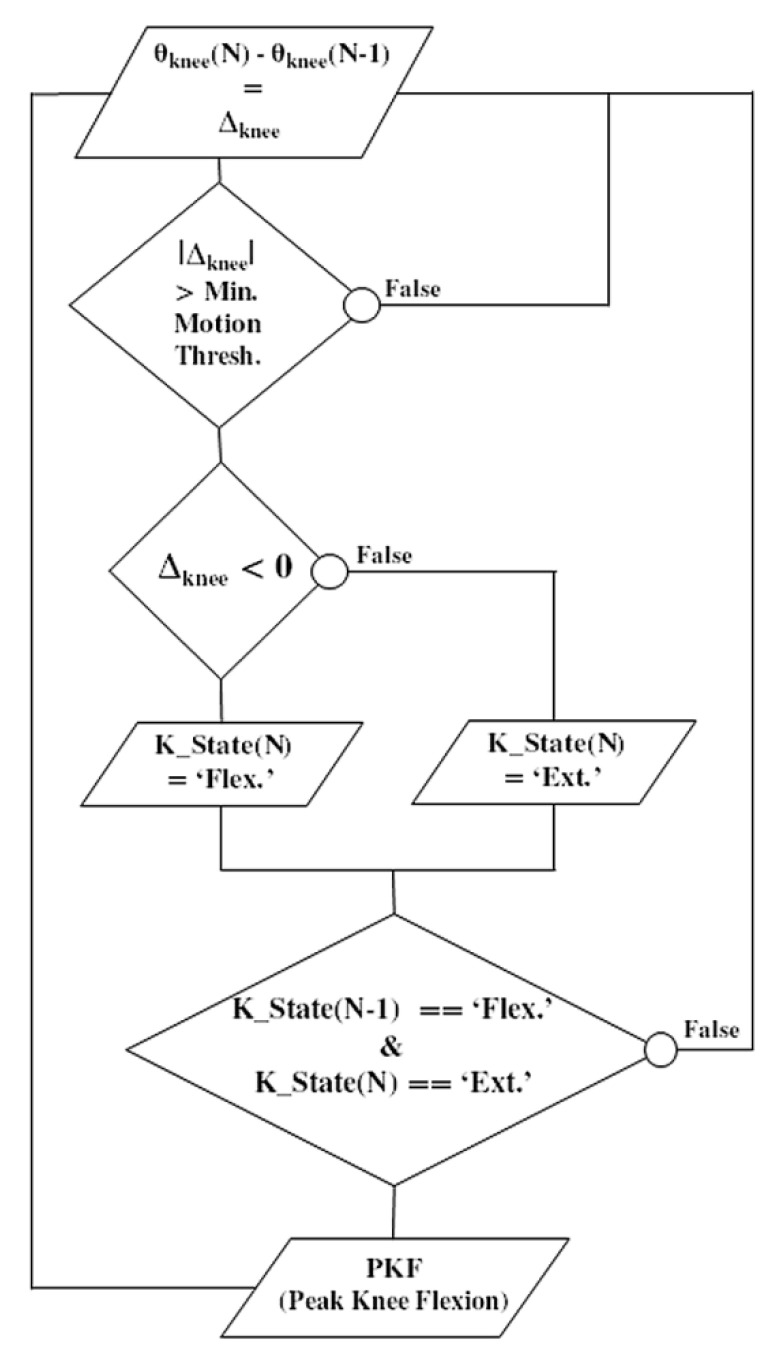
Flowchart illustrating the real-time detection algorithm of the ‘peak knee flexion’ (PKF) as a pre-stance stimulation event. Knee angle gradient Δ_knee_ is computed at each new data (N). If the knee angle variation is above a minimum preset threshold, a motion is considered. The sign of Δ_knee_ enables to know if the knee has been flexed or extended. If the previous state (K_state) was flexed and the current one is extended, a PKF flag is risen.

Meanwhile, this pre-stance event is not adapted to all pathological gait patterns. It can lead to false positives when the gyrometer waveform does not show any detectable characteristics, or when the dynamic range of the gyrometer is not adapted to a slow gait pattern. In this case, the knee flexion angle can be considered as an alternative. Over the gait cycle, the knee angle is a smoothed signal, less sensitive to noise, vibrations or dynamic of motion. In addition, it presents an interesting waveform characteristic: the peak knee flexion (PKF) angle. The PKF angle corresponds to an event about 14% earlier than the terminal swing (TS) [25]. In order to reliably detect in real time this event from the knee angle, a specific PKF detection algorithm was developed (Figure 5). The principle is to monitor the knee angle gradient Δ_knee_ at each new data (N). If the knee angle variation is above a minimum preset threshold, then a motion is considered. Monitoring the sign of Δ_knee_ enables to know if the knee has been flexed or extended. If the previous state (K_state) of the knee was flexed and the current one is extended, a PFK flag is risen.

## 3. Clinical Protocol

### 3.1. Subjects

Following a supratentorial ischaemic or hemorrhagic stroke (ICD-11 8B20), the subjects should be able to walk on a restricted perimeter (<50 m) without human help, with or without a walking assistive device (e.g., tripod, walking stick).

Eleven participants were involved in this study (mean 56.7 ± 7.5 years old; five females). 40% had a right sided paresis. One subject was excluded because electrical stimulation was causing too much discomfort.

The protocol was approved by a national ethical committee (#RCB 2017-A03611-52), all subjects provided informed consent prior to the experiments. Experiments were carried at CRF La Châtaigneraie rehabilitation center (Menucourt, France).

### 3.2. Protocol Conduct

#### 3.2.1. Balance Training Protocol

**Figure 6:**
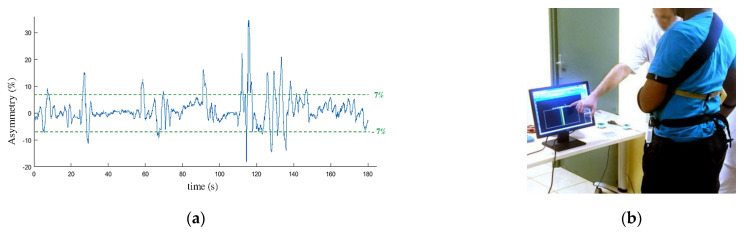
Balance training exercise: each participant was asked to maintain his balance to an asymmetry below 7% (**a**) of the total weight during 180 s with the help of a visual feedback (**b**).

Balance training exercise was initially performed in order for the participants to get used to support their weight on their paretic limb. The participants were equipped with the pressure insoles and standing in front of a screen. They were asked to maintain their body sway under 7% of asymmetry (expressed as a percentage of their total weight measured by the insoles) with the help of a visual feedback (Figure 6). Based on Mizrahi et al. study [26] performed on able bodied subjects, we considered the ±7% range as a reference value to reach for a normal body balance. The total time spent in the ±7% range was calculated over a 180 s session in order to quantify the progress of the participant and his ability to follow the walking protocol.

#### 3.2.2. Walking Protocol

Following the balance training session, participants were asked to walk on a flat floor over 16 m starting from a standing position. The first 3 m aimed at achieving a steady state gait on the following 10 m. The last 3 m enabled the participants to slow down and stop. Only the 10 m of steady state walking were considered for post-processing and results. An oral instruction was given at the beginning of each trial to encourage the participants to transfer their weight onto the paretic leg.

Three different walking conditions were considered:

- Condition 1 (C1): no assistance, the participants walked at a self-selected speed.
- Condition 2 (C2): the paretic limb was equipped with a knee orthosis (Orliman©, Rennes, France) limiting the knee flexion angle and the knee extension angle around 5°.
- Condition 3 (C3): the paretic limb was stimulated following the control modality described in this study.

The three conditions were successively repeated in a random order, in order to avoid a possible learning effect, until at least three trials of each condition were successfully recorded or until the participant’s fatigue prevented him/her to further continue the experimental protocol.

### 3.3. Evaluation Criteria

The first main criterion was computed following the method described by Patterson et al. [27] as the symmetry ratio of stance time between paretic and non-paretic limbs. The second main criterion was computed as described by Mizrahi et al. [9] as the asymmetry in weight bearing during the stance phase, expressed in percentage of the total weight. These two main criteria aimed at quantifying both in time and magnitude the gait asymmetry using the foot pressure insoles data. The secondary criteria aimed at measuring the comfortable walking speed and quantifying gait quality and physiological cost through a series of different markers. Perceived effort of walking using a Borg scale [28] from 6 (no exertion) to 20 (maximum exertion) was evaluated.

Physiological cost index (PCI) was calculated as the ratio of the difference in working and resting mean heart rates (bpm) and the self-selected (comfort) walking speed (m/min). The *PCI* value reflects the energy expenditure for walking and is expressed as heartbeats per meter by Mc Gregor’s equation [29]:

(2) $$PCI=\frac{\underline{HR_{work}}-\underline{HR_{rest}}}{Walk_{speed}}$$

In order to accurately and automatically compute the PCI between each experimental trial, two infrared passage detection systems were installed at the beginning and at the end of the 10-m experimental path and synchronized with the heart rate monitor armband via a data acquisition device.

**Figure 7:**
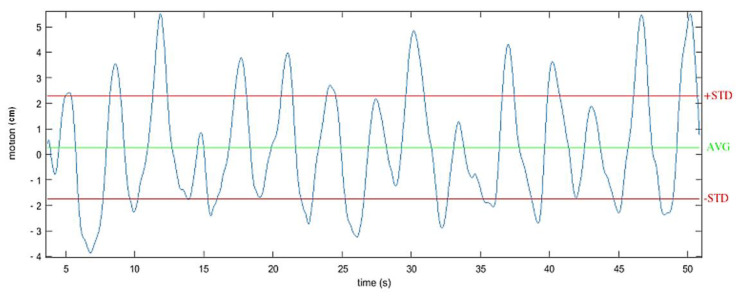
Example of one recording of the vertical displacement of the center of mass during walking. Estimated by double integrating the filtered acceleration from an IMU located at the second sacral vertebra level.

The vertical displacement of the center of mass (CoM) was also measured. Indeed, several studies have demonstrated the correlation between the vertical displacement of the trunk during gait and the associated physiological cost in able bodied and SCI individuals [30]. Enomoto et al. [31] showed that the vertical movement of an inertial sensor mounted on the sacrum was equivalent to the vertical movement of the center of mass. For this protocol, the vertical displacement of the center of mass was then estimated by double integrating the acceleration recorded on the vertical axis of the IMU located at the back of the subjects. Based on previous works [15], the vertical acceleration component was determined using a sensor fusion algorithm, filtered using a forward-backward highpass Butterworth filtering (order 1, Fcutoff = 0.001 Hz) and updated at each stride (zero velocity update). The considered value corresponded to twice the standard deviation around the mean estimated displacement (Figure 7) along the 10-m experimental path.

## 4. Results

The following analysis and results refer to 10 subjects. A descriptive analysis of data was performed comparing means (Mean), standard deviations (SD), medians (Med), quartiles (Q) and ranges taking into account all the trials (Table 1).

An analysis of variance (ANOVA) was then performed on main and secondary evaluation criteria (Table 2). The conditions for applying the regressions were checked graphically on the residuals (normality and heteroskedasticity).

No significant *p*-value was found on main evaluation criteria but the *p*-value regarding the trunk vertical motion was found to be significant, with a value of *p* = 0.0089.

To compare combined effects of walking conditions on trunk vertical motion, Tukey tests were performed. Raw *p*-values were corrected with a Holm correction due to the multiplicity of the tests. A corrected *p*-value of 0.038 was computed regarding the comparison between Conditions 3 and 1 (Table 3). This means that Condition 3 has a significant effect on trunk vertical motion.

## 5. Discussion and Conclusions

Being able to finely control the knee angle could play a key role in gait recovery of people with CNS disorders. Meanwhile, most of the studies using FES have mainly focused on correcting the drop foot syndrome. Few studies have been able to overcome the constraints of an ambulatory FES closed-loop control system in order to investigate the rehabilitative potential of such a modality. Our study aimed at developing a sensor-based multichannel FES system to control knee joint while taking into account the numerous related challenges. The objectives were to demonstrate the feasibility of using such a system as a rehabilitation device and to investigate in post-stroke gait the effects of FES on stance phase asymmetry. From a technical and analytical point of view, through an elaborated hardware and algorithmic architecture, the control strategy successfully triggered as intended the stimulation during the gait, depending on the decisional algorithm executed in real-time. In further studies, a desirable improvement would consist in adding a wireless electromyography system to accurately monitor the overall latency of our execution chain until muscle force response.

No significant clinical outcomes have been observed on the main criteria: asymmetry of stance time and weight bearing. This could be partly explained by the fact that no participants received an electrical stimulation training period before the walking protocol. Such a training would have been too difficult to standardize in a heterogeneous population without inducing a bias. On the other hand, no prior training enabled subjects to be considered as naive in regards with FES in order to better judge its interest. Most of the protocols using FES as a gait rehabilitation modality rely on longitudinal studies performed over a long period of time involving repeated observations, where the participants can get used to walk with FES on longer distances. Choosing the relevant secondary criteria was an important point to properly evaluate the outcomes of our protocol on a short distance without prior training. In our case, the rationale to include PCI when only walking 10 m could have been discussed. Some researchers [32,33] measured heart rate during a 10-m walk before and after training with a peroneal stimulator in chronic stroke patients. They reported a reduction in the PCI of 31% with the stimulator-based therapy. However, Hood et al. [34] noted that this PCI calculation was based on an insufficient time to reach a steady state of heart rate and oxygen consumption, as required in the definition of the PCI. Other studies suggested that a floor-walking track offers a more functional medium than a treadmill for assessing the PCI in elderly subjects [35,36].

**Figure 8:**
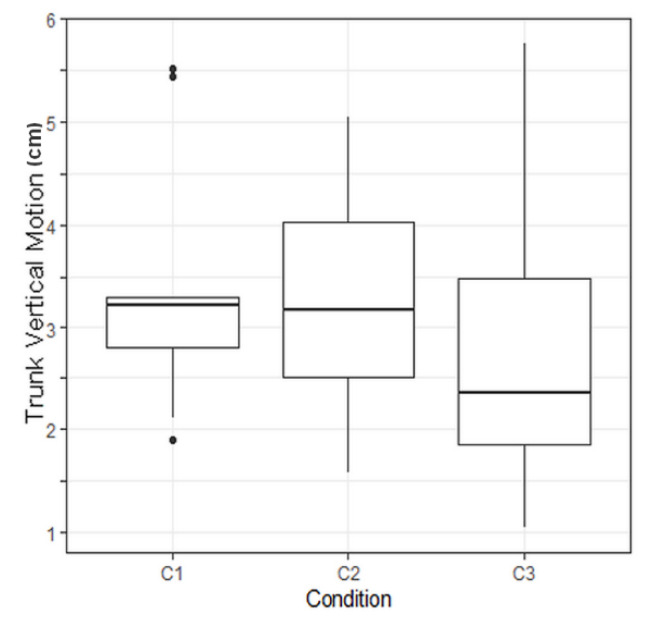
Boxplot illustrating the trunk vertical motion depending on the protocol condition. C1: no assistance, C2: with knee orhosis, C3: with electrical stimulation. C3 led to a significant reduction of the trunk vertical motion.

A *p*-value of 0.038 was computed regarding the effect of C3 modality versus C1 modality on trunk vertical motion. This result means that FES made this evaluation criterion significantly vary compared to gait without assistance (Figure 8). Due to the limited amount of data, the clinical relevance of this result has still to be demonstrated.

Despite the fact the main criteria were not improved by FES, the vertical displacement of the trunk has been shown to be a relevant marker of gait quality and physiological cost during gait [37]. As a preliminary study, this marker could be used for calculating the number of participants to include in a future larger randomized trial. As observed in Saini et al. [38], the method used to estimate the trunk vertical motion could highly affect the results and conclusions. In a future study, this criterion could be measured using an optoelectronic measurement system in order to reduce possible errors compared to an IMU based estimation.

This preliminary work has led to promising technical and rehabilitative potentials of a sensor-based multichannel FES system in post-stroke gait where the use of FES to control knee joint could significantly improve gait quality. A longitudinal study with repeated observations on a long period would be required to positively affect the other evaluation criteria used in this study and better assess the relevance of the clinical outcomes. The algorithms and hardware architecture investigated could be also applied to other CNS related gait disorders. If evidence for FES as a treatment option in adults has been widely observed over the last 30 years, the clinical knowledge of such therapy in children and young people still needs to be addressed [39]. Despite promising results in children suffering from cerebral palsy [40], technological issues and the lack of devices adapted to a pediatric population could partly explained the low number of studies in this field. If widely demonstrated, the use of FES could have a strong research and clinical impact on this population and be considered as a serious alternative to surgery. Our sensor-based multichannel FES system overcomes numerous technological limitations and provides a useful tool for the clinical researchers. It could open the way to new rehabilitative approaches and lead to new applications, not only in post-stroke gait adult rehabilitation but also in children with cerebral palsy.

## Figures and Tables

**Table 1:** Subjects description averaged on all trials, quantitative variables (n = 70).

	Mean +/− SD	Med [Q1; Q3]	Min; Max
Gait Velocity (m/min)	18.6 ± 7.4	16.5 [12.7; 25.3]	7.8; 34.5
10 m path duration (s)	37.7 ± 15.5	36.3 [23.7; 46]	17.4; 76.8
PCI	0.3 ± 0.8	0.4 [0.1; 0.8]	−3.2; 2.2
Borg	9.2 ± 1.7	9 [8; 10]	7; 13
Gait quality	3.8 ± 0.7	4 [3; 4]	2; 5
Stance Force Index (%)	−36.0 ± 32.3	−41.4 [−55.2; −4.6]	−90.5; 21.3
Stance Force Ratio (%)	0.7 ± 0.2	0.7 [0.6; 1]	0.4; 1.2
Stance Time Index (%)	−19.1 ± 11.9	−20.1 [−27; −14.7]	−39.2; 23.9
Stance Time Ratio (%)	0.8 ± 0.1	0.8 [0.8; 0.9]	0.7; 1.3
Stance time left foot (s)	1.7 ± 0.6	1.8 [1.3; 2.2]	0.8; 2.9
Stance time right foot (s)	1.7 ± 0.6	1.6 [1.1; 2]	0.9; 3.4
Trunk vertical motion (cm)	3.3 ± 1.6	2.8 [2.1; 4.1]	1; 9.9

**Table 2:** ANOVA results.

Variable	*p*-Value
Stance force index	0.33
Stance force ratio	0.078
Stance time index	0.095
Stance time ratio	0.16
Gait velocity	0.31
Path duration (log.)	0.36
Borg (log.)	0.14
PCI	0.35
Gait quality	0.78
Trunk vertical motion	0.0089

**Table 3:** Tukey tests: comparison of combined effects of walking conditions on trunk vertical motion.

	Condition 2		Condition 3	
	Raw	Corrected	Raw	Corrected
Condition 1	*p* = 0.59	*p* = 0.59	*p* = 0.013	*p* = 0.038
Condition 2			*p* = 0.030	*p* = 0.060
